# Developing Intelligent Robots that Grasp Affordance

**DOI:** 10.3389/frobt.2022.951293

**Published:** 2022-07-05

**Authors:** Gerald E. Loeb

**Affiliations:** Department of Biomedical Engineering, University of Southern California, Los Angeles, CA, United States

**Keywords:** haptics, exploration, dexterity, manipulation, reflexes, learning

## Abstract

Humans and robots operating in unstructured environments both need to classify objects through haptic exploration and use them in various tasks, but currently they differ greatly in their strategies for acquiring such capabilities. This review explores nascent technologies that promise more convergence. A novel form of artificial intelligence classifies objects according to sensory percepts during active exploration and decides on efficient sequences of exploratory actions to identify objects. Representing objects according to the collective experience of manipulating them provides a substrate for discovering causality and affordances. Such concepts that generalize beyond explicit training experiences are an important aspect of human intelligence that has eluded robots. For robots to acquire such knowledge, they will need an extended period of active exploration and manipulation similar to that employed by infants. The efficacy, efficiency and safety of such behaviors depends on achieving smooth transitions between movements that change quickly from exploratory to executive to reflexive. Animals achieve such smoothness by using a hierarchical control scheme that is fundamentally different from those of conventional robotics. The lowest level of that hierarchy, the spinal cord, starts to self-organize during spontaneous movements in the fetus. This allows its connectivity to reflect the mechanics of the musculoskeletal plant, a bio-inspired process that could be used to adapt spinal-like middleware for robots. Implementation of these extended and essential stages of fetal and infant development is impractical, however, for mechatronic hardware that does not heal and replace itself like biological tissues. Instead such development can now be accomplished *in silico* and then cloned into physical robots, a strategy that could transcend human performance.

## 1 Defining Intelligence

The term “artificial intelligence” (AI) implies a definition and criteria for what constitutes intelligent behavior. Alan Turing’s “imitation game” (Turing, 1950) was an attempt to objectivize a debate that was already well underway at the dawn of electronic computing, which Turing concisely critiqued. Most of Turing’s predictions about the capabilities of computing machines have been met or greatly exceeded, including playing complex strategy games such as chess and go, recognizing written and spoken language, and identifying objects in complex visual scenes.

Most of the AI successes mentioned above were obtained by abandoning the design of symbolic reasoning algorithms to solve specific problems in favor of imitating, at least in part, the associative learning rules used by biological neurons (Hebb, 1949). That strategy started only a few years later with the Perceptron (Rosenblatt, 1958) but lagged behind until its much greater demands on computing power could be fulfilled (Hinton, 2012). At least for those who eschew mind-body dualism, there is no fundamental reason why a sufficiently large and accurate simulation of the human nervous system could not be as intelligent as a human being. And yet most participants and observers of AI have the nagging feeling that its current shortcomings are not just quantitative but also qualitative.

Rather than evaluating intelligent machines by what they can do, it may be more fruitful to consider how they fail. We are familiar with and expect to accommodate human failings, but AI failings appear to be qualitatively different. The problem is most easily appreciated by so-called “adversarial attacks” on deep-learning neural networks (NNs), which will confidently identify previously seen objects when confronted with carefully designed but apparently nonsensical visual patterns (Goodfellow et al., 2014). These errors are nothing like the many optical illusions to which humans are prone (Coren and Girgus, 2020). Language translation has become quite good at dealing with declarative sentences but makes egregious mistakes when contextual judgment is required (Läubli et al., 2020). Similar but less well-understood failures occur when industrial robots interact with objects in circumstances that differ only slightly from those for which they have been well-trained.

The various, different examples of failure above have in common a lack of understanding of the fundamental nature and relationships of the things that the machine is identifying or manipulating. Learning to classify objects according to arbitrary properties that tend to recur in those classes provides no information about why those properties are (or are not) important to the class, why they occur together or how their combination results in the emergent properties of the object (recently discussed in *The Economist*, “AI for vehicles—is it smarter than a 7-month old?” 4, September. 2021, pp. 65–66, and *IEEE Spectrum,* “Why Is AI So Dumb?” October. 2021, pp. 24–62.) From where does such understanding come, if not in lengthy training on increasingly large datasets?

## 2 Intelligent Sensorimotor Systems

**FIGURE 1:**
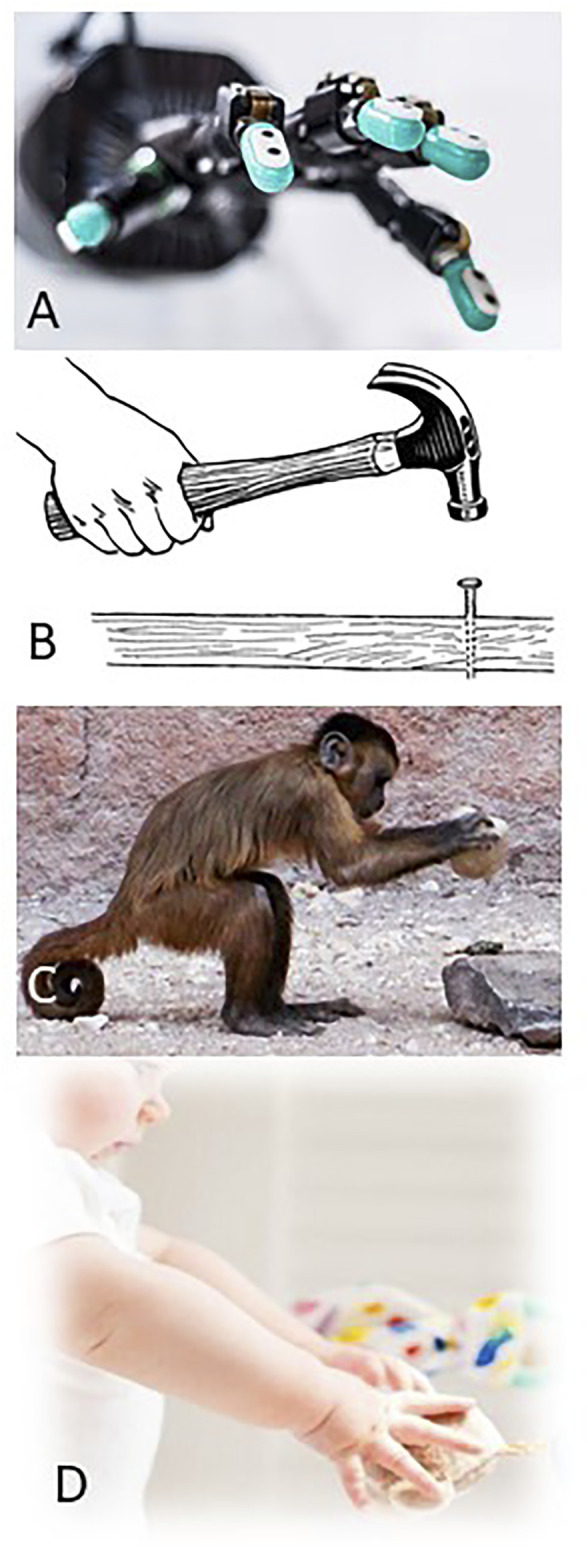
**(A)** Shadow Robot^®^ hand equipped with BioTac^®^ sensors. **(B)** Training example of human hammering a nail. **(C)** Macaque demonstrating the affordance of using a rock as a hammer. **(D)** Infant learning how to explore and categorize objects.

This review compares and contrasts the various sensorimotor systems illustrated in Figure 1, all of which are capable of or aspire to intelligent behavior. It focuses on the creative haptic behavior captured by the psychological concept of “affordance” (Gibson, 1977). Objects in our environment are important not because we have learned their names (declarative memory) but because they can be used to accomplish tasks. We can imagine what task an object affords even when we have not previously used the object for this purpose and when the manner in which the object must then be used may be different from procedural memory based on prior training on the task. Affordance and the related concept of causation presumably depend on both declarative and procedural memory but they require insights that must be computable from the structure of such memories.

**FIGURE 2:**
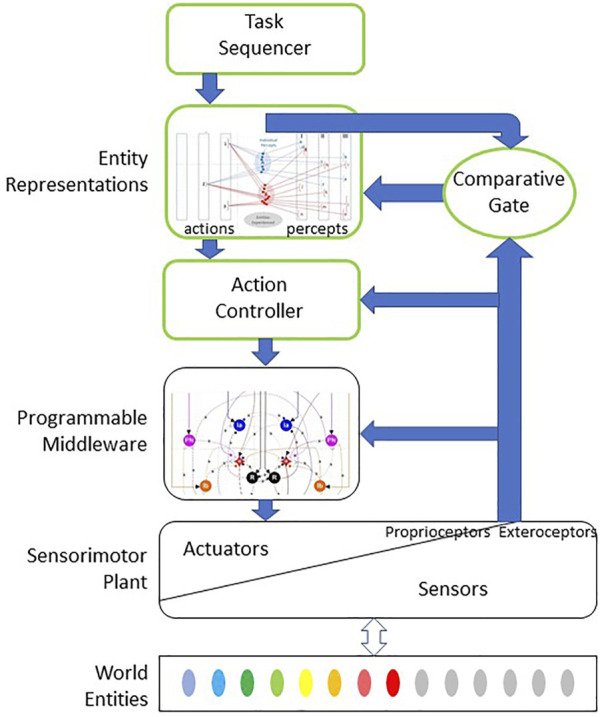
Bio-Inspired theory of computation for haptic performance whereby a sensorimotor plant is used to explore, identify, classify and use various entities in the external world. Exploratory and manipulative actions are coordinated and regulated by programmable middleware in subcortical motor pathways that self-organize starting during fetal development (spinal cord and deep cerebellar nuclei in vertebrates; see Figures 5, 6). Blocks outlined in green represent cortical executive for those actions, which uses stored neural representations of external entities consisting of learned associations between exploratory actions performed on and sensory percepts obtained with such entities (see Figure 3). During an exploratory or manipulative action, the cortical controller compares the actual sensory signals to those expected if the entity is the currently most probable one based on previous experience. If no agreement can be obtained with any previously experienced and stored representation, the sensory data are admitted to the cortex and saved as part of the representation of a new category of entity.

This review proposes a system architecture (Figure 2) that could self-organize through experience to recognize and utilize affordances similarly to a human, the gold standard for intelligent behavior. It is based on an internal representation of objects as the experienced associations of actions with percepts (next section). Developing such a representational system from scratch as an infant appears to require millions of exploratory movements, which an infant learns to make in a graceful and efficient manner using self-organizing coordination circuitry in the spinal cord (Section 7). This lengthy process is akin to system identification and is probably impractical to fulfill with mechatronic robots but could be met with software simulations of them.

## 3 Intelligent Exploration

Understanding rather than simply observing the world may emerge through the processes of “interactive perception,” recently reviewed by (Bohg et al., 2017). The history of active perception in computer vision was recently reviewed by (Bajcsy et al., 2018). The robotic challenges are usually presented from the perspective of locating and identifying a given, desired object in a cluttered scene or quantifying a particular property of a given object (e.g., weight or hardness) or environment (e.g., traversability) (Jie et al., 2010). By contrast, interactive perception in animals is necessary for animals to discover the existence and behavior of objects in a world about which they initially know nothing, as in kittens learning to navigate based on their visual experience (Held and Hein, 1963). The real physical world and an agent’s interactions with it have many properties that are incompatible with currently available machine-learning algorithms (Roy et al., 2021).

Tactile sensory information cannot even be obtained, much less interpreted usefully, without interaction between fingers and objects, both for humans (Katz, 1925; Klatzky and Lederman, 2003) and for robots employing artificial tactile sensors (Wettels et al., 2013). But this begs the questions of how do humans and how should machines decide which interactions to generate when exploring an unknown object to identify it. Mathematically inverting Bayes’ theorem (Bayes and Price, 1763) allows computation of which possible exploratory action and observation will be most useful next (Fishel and Loeb, 2012), given the current probabilities that resulted from the preceding observations (Bayesian priors). Briefly, for each possible next exploratory action, the Bayesian prior probabilities are used to weight the confusion matrix of sensory signals resulting from such previous exploration of all possible objects. The exploratory action with the lowest weighted sum of its matrix is the optimal next action to perform.

**FIGURE 3:**
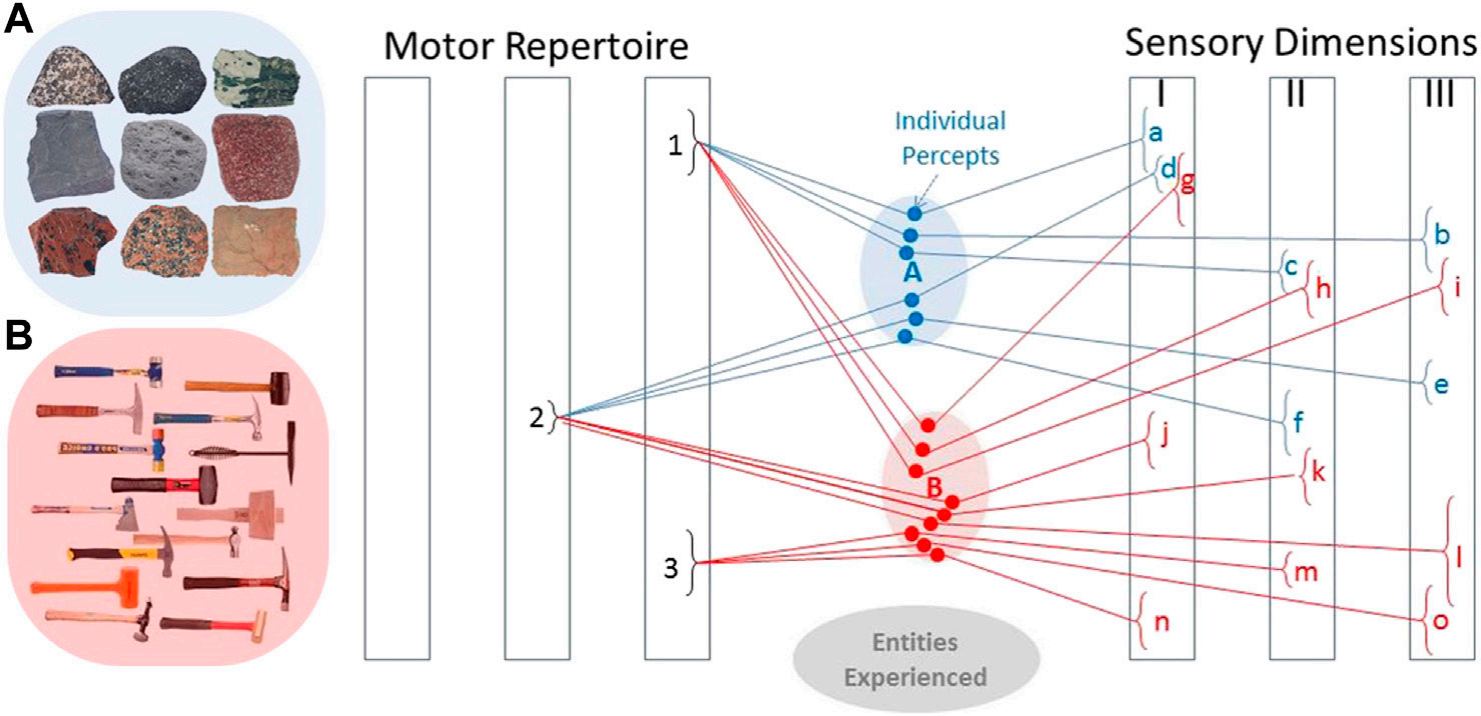
The internal representation of entities in the brain are percepts consisting of sensory experience during selected motor actions. These tend to cluster for entities that we come to see as similar (e.g., rocks in cluster **(A)** and are distinct in at least some dimensions for entities that are different (e.g., hammers in cluster **(B)**. Given such an associative memory, it is possible to see whether a given percept will likely discriminate between currently probable alternatives for an entity’s identity, as well as to look up the motor action that gives rise to a desired pattern of sensory feedback when manipulating an identified entity. Adapted from (Loeb and Fishel, 2014).

The above algorithm for Bayesian exploration was used to build a machine that could identify objects according to their textures. It was extraordinarily accurate and efficient and eerily humanlike in its deliberations (Fishel and Loeb, 2012). It was tested initially on 117 flat, homogeneous materials explored by three stroking movements (different velocities and forces) and observed on three perceptual axes, resulting in nine dimensions. Repetitive measures had a standard deviation of ∼3%–5%, resulting in at least 10 distinguishable levels on each axis (illustrated graphically in Figure 3). Any reasonably complete neural network training set for such a hyperspace would have required millions of samples, but the iterative strategy of Bayesian exploration appears to overcome this “curse of dimensionality” (Bellman, 1957). It achieved 95% accuracy with a median of five exploratory movements of unknown samples from the training set. A commercial version of this machine (Toccare^®^ from SynTouch Inc.) expanded characterization to five stroking and poking actions that quantified 15 perceptual dimensions including various aspects of texture, friction, mechanical deformation and thermal flux. After characterizing 500 materials (a very sparse representation of a very large hyperspace), it achieved 98% identification accuracy with a median of three exploratory movements (Fishel, 2017). Unlike purely perceptual challenges such as visual object identification, cost of search (time, energy and risk of damage) in physically embodied systems cannot be ignored. We value human experts not just because they are accurate but also because they are efficient.

High dimensionality and sparseness of experience are advantageous for the Bayesian exploration strategy. If confronted with a novel object not previously characterized, Bayesian exploration will identify the closest previously experienced object and the probability of a match, which if below acceptable could be used to trigger the creation of a new internal representation for the novel object (Loeb and Fishel, 2014). This creative step is essential to develop from scratch an internal representation and classification of whatever objects happen to have been experienced, but it remains to be implemented and tested algorithmically.

An infant makes tens of millions of trial exploratory movements as it learns how to achieve desirable outcomes with its limbs and their interactions with objects (Piek, 2006). Neural networks tend to self-organize around recurring patterns by increasing synaptic strength among signals that tend to be frequently associated (Hebb, 1949). If those experiences include both the motor commands and the resulting sensory information, the internal representations will associate the similar experiences that arise from interacting with the available objects (Loeb and Fishel, 2014). Such clusters of experiences presumably provide the basis for the abstractions that we eventually associate with learned, categorical words such as “rocks” and “hammers” (Figure 3). Biologically realistic neural networks can function as an associative memory (Baum et al., 1988; Gerstner, 1990; Lansner, 2009; He et al., 2019) and can compute Bayesian probability (Mcclelland, 2013).

When we encounter objects that are insufficiently similar to the categories that we have already formed, an executive function must evaluate the degree of mismatch and then decide to treat those experiences as a separate cluster reflecting a newly discriminable entity. This is the function of the “Comparative Gate” element in Figure 2, which compares the actual sensory data obtained during an exploratory action with the memory of the comparable sensory data associated with the entity that is currently the most probable Bayesian prior. If they agree, the real sensory data can be discarded and the brain can proceed to another action. If they disagree and no other previously experienced entity is consistent with the cumulative actions and perceptions, the real sensory data should be stored as part of the internal representation of a new entity. One candidate for the comparative gate component would be the thalamocortical loop (Halassa and Sherman, 2019). The system architecture also requires motivational and integrative components not illustrated so that the tolerable level of uncertainty about an entity’s identity can be adjusted to avoid acting precipitously in unfamiliar and potentially dangerous situations (Roy et al., 2021). These might reasonably be performed by the biological basal ganglia projections to thalamus, for which computational neural network models are starting to be developed (Hazy et al., 2007).

## 4 Discovering Causality

The process of recalling a previously experienced object based on active exploration is more than a simple association of remembered actions and percepts. The linkages in Figure 3 are causal as well as associational. The ability to interact with objects sets the brain on the course of abstracting principles that might underlie such interactions rather than storing simple memories of the interactions themselves. As noted in the introduction, the many perceptual illusions to which humans are prone are completely unlike the failings of AI. They suggest that humans do not compare newly received sensory data to a bank of remembered sensory data. Instead they abstract experiences into presumed causal mechanisms that then allow them to reconstruct expected sensory data and compare it to newly received sensory data. Over time, such causal abstraction gets deeper and better at reconstructing what appear to be memories but are actually illusions. If the illusions are similar enough to newly received sensory data, we dismiss the differences and accept as fact the conjured illusion. The causal abstraction need not make any physical sense; it simply needs regenerative power. The nonsensical tricks that people are taught to improve their memory for arbitrary lists and names are an example of this (Bower, 1970).

One long-known failing of artificial neural networks is “catastrophic forgetting” of previously recognized entities after synaptic weights are modified to represent more recently experienced entities (French, 1999). Biological organisms achieve “lifelong learning” by a variety of mechanisms, some of which have been applied in AI (Kudithipudi et al., 2022). Learning principles of causation instead of memorizing experiences may avoid the problem in the first place. The incoming sensory data would be compared to the predicted data and discarded if sufficiently congruent, avoiding any plastic drift of the neural network. Neuromorphic algorithms to achieve this have yet to be developed, however.

How many principles of causation we discover and the categories of entities that we create depend on the entire experiential history of individuals. How we might treat borderline objects that don’t behave according to existing categories depends on the perceived value of drawing fine vs. coarse distinctions, which also differs among individuals based on experience and perhaps underlying personality differences. As a result, one person’s internal representations of objects are likely to be different from another’s even if they have all learned to use the same words for those representations. Humans differ in their abilities to recognize causation and anticipate outcomes, depending on the richness of their experiences exploring and manipulating objects. Programming or teaching robots without such active experiences is likely to lead to types of failures that humans generally avoid. Ugur et al. (2015) demonstrated the utility of prior simple but active experience with objects when humans were asked to train a robot to perform a complex sensorimotor task with those objects.

## 5 The Emergence of Affordances

The database for the material identification machine was based on discrete test objects with known identities that were explored according to algorithmically defined movements and percepts. An infant, however, must develop all this from scratch (Loeb et al., 2011). In so doing, the infant develops a much richer representation of the world than the simple one implied by declarative memory. It includes the active imagination of what might happen next, a process that has been identified neurophysiologically in so-called “mirror neurons” (Loeb and Fishel, 2014; di Pellegrino et al., 1992; Oztop et al., 2013).

**FIGURE 4:**
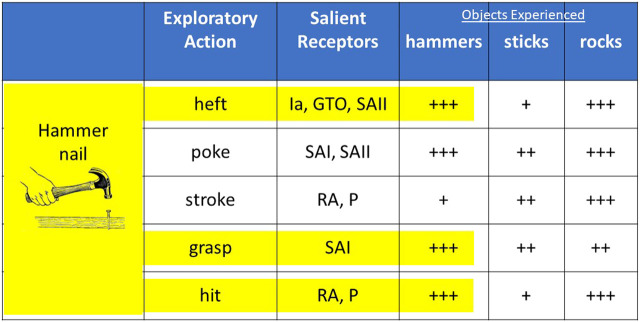
Suppose that an individual has previous experience exploring various hammers, sticks and rocks and then learns the high-level task of driving a nail that includes a subset of those exploratory actions while using a hammer (highlighted in yellow). In the absence of a hammer, the previously experienced entities that produce the most similar sensory activity (+s indicate relative strength) for the relevant actions will be rocks, which afford driving a nail. (Ia = spindle primary afferent; GTO = Golgi tendon organ; SAII = slowly adapting,skin-stretch receptors; SAI = slowly adapting, normal force receptors; RA = rapidly adapting vibration receptors; P=Pacinian corpuscles).

Suppose that we have learned to use a previously categorized object to perform a task such as using a hammer to drive a nail. This task involves combinations of some actions that we have previously taken with the familiar object while excluding others (Figure 4). This will create new, higher level associations that are our procedural memory for the entire experience of driving a nail. If we then find ourselves needing to drive a nail without a hammer, we can query our associational memory for the class of objects that produced the most similar percepts for the actions relevant to driving a nail with a hammer. This would be rocks in the associational database represented in Figure 3. Then we can find the association between the specific motor actions that led to those percepts when using such an object, which would include the enclosure grip that we have learned for rocks rather than the power grip suitable for hammers. We have grasped the concept of affordance.

It is worth emphasizing that conventional divisions such as declarative memory to identify objects and procedural memory for sequences of action would not enable a human or a machine to realize affordances. Percepts are useful only in the context of the actions that caused them; skills consist of sequences of actions accompanied by percepts that are part of the procedural memory. Appreciating affordance requires continuously integrating well-chosen exploratory actions and perception during all tasks. Affordances are inherently insights into fundamental properties of objects rather than simple generalizations across similar perceptual experiences.

An extensive, recent review of the concept of affordance and its applicability to robotics concluded that it was a promising approach to improve motor capabilities, especially when interacting with unfamiliar objects (Jamone et al., 2018). Procedures and algorithms to acquire such information autonomously, however, are still limited to specific properties of individual objects (e.g., graspability, liftability, pourability) rather than the ability to generalize and innovate that is associated with biological affordance. Various algorithms have been proposed to program robots with knowledge of affordances associated with objects, but the autonomous discovery of such relationships through exploration and experience that enables human dexterity remains without an overarching theory of computation or a practical platform to implement it in robots.

## 6 Actively Intelligent Robotic Systems

There is no fundamental reason why artificial intelligence could not operate a sufficiently dexterous and sensitive robot to achieve the same knowledge and anticipation of the world and the recognition of affordances that are achieved by young children and macaque monkeys. There are, however, substantial technical challenges which, if overcome, might offer substantial enhancements over human performance (Table 1).

**TABLE 1:** Comparison of biological and robotic strategies for meeting requirements of intelligent systems.

Requirement	Biological Strategy	Robotic Challenge	Robotic Strategy	Robotic Advantage
Multimodal sensors	Dispersed	Wiring	Telemetry	BW and SNR
Robust sensors	Regenerated	Vulnerable	Modular	Serviceable
Experiential	Infancy	Wear and Tear	Model-based	Scaleable
Adaptive	Heterogeneity	Unpredictable	Selection	Cloneable
Iterative	Graceful	Fairing	Middleware	Speed

### 6.1 Multimodal Sensors

Unlike robotics engineers, Mother Nature puts more emphasis on the sensory signals coming from limbs than the motor commands going to them. During normal use, the data rate from all proprioceptors is 10–50 times greater than for the command signals to the muscles in which they reside. Most limb muscles have about the same numbers of each of spindle primary and secondary afferents and Golgi tendon organs as they do motor units (∼100–500), but all the sensors tend to be active at rates of 50–150 pulses per second whereas most tasks require fewer than half the motor units firing at 10–30 pulses per second. The thousands of specialized cutaneous receptors in the glabrous skin of a primate hand provide even more sensory information when manipulating objects. Mechatronic transducers can be made quite small and low-powered using application-specific integrated circuits (ASIC) and microelectromechanical systems (MEMS) technologies (Newell and Duffy, 2019) but providing power and data links through wire harnesses and connectors rapidly becomes impractical in a complexly moving limb. If this problem were overcome *via* bidirectional telemetry and energy harvesting (Guo et al., 2017), robotic sensors could have a substantial advantage over biology in signal-to-noise ratio, bandwidth and latency of sensory transmissions. In contrast to engineered sensors, biological mechanoreceptors tend to have limited, nonlinear dynamic range, substantial noise and nonorthogonal modalities. Their numbers compensate for their individual limitations but this complicates extraction of state feedback (Scott and Loeb, 1994).

### 6.2 Robust Sensors

Large numbers of tiny mechanical sensors distributed in moving structures that interact forcefully with objects will eventually become damaged. Mother Nature constantly regenerates such mechanoreceptors, a trick not available to engineers. Wireless power and communication would greatly facilitate modular sensor replacement at service intervals, thus providing an advantage over organisms that recover slowly and often incompletely from large or repetitive injuries.

### 6.3 Experiential Learning

Replicating the tens of millions of exploratory movements of an infant would take years for a mechatronic robot while wearing it out. Computer models of robots (Ivaldi et al., 2014) and even their tactile sensors (Narang et al., 2020) are starting to be accurate and fast enough to permit learning in virtual environments at hyperspeeds. Such learning is scaleable so that many robotic controllers can be trained on different combinations of objects and environments, including those that may not be available physically such as unusual gravitational fields. Transfer of learning from simulated to physical robots is now being applied to the problem of robust and efficient locomotion (Hwangbo et al., 2019). Some of the challenges to creating valid virtual training environments for haptics were discussed by (Bajcsy et al., 2018). This tactic is somewhat analogous to the transition of perceptual AI from writing bespoke algorithms to training large and deep neural networks, suggesting that it is important and possible but will not be easy.

### 6.4 Adaptive Representations

The internal representation of the world that results from incremental experience is inevitably idiosyncratic rather than predictable or standardized. Human societies are organized to distribute various tasks among individuals to take advantage of their natural heterogeneity, whereas industrial robots need standards and quality control. The robotic advantage for scaleable virtual learning can be further exploited by cloning the best robotic controller that has emerged from training, including both hardware and software. Perceptual AI based on genetic algorithms and deep-learning neural networks is already doing something like this for commercial applications (Tian et al., 2018). Even if a human’s genes could be cloned, each cloned individual would still require many years of training, so the result would still be somewhat heterogeneous.

### 6.5 Rapid Iteration

What we call human dexterity consists of remarkably rapid and smooth transitions between actions that are simultaneously explorative and executive. Robots, by contrast, tend to separate those activities into discrete movements with sudden starts and stops. High-level controllers can explicitly compute overall trajectories that minimize jerk (Valente et al., 2017) but only if they know the sequence of movements in advance. Iterative Bayesian exploration of objects was successful and efficient for haptic identification of objects by a robot, but it also took much longer to execute than a human performing similar exploratory movements, despite the faster and more controllable motors of the robot. Humans “fair” their iterative exploratory movements and extract useful sensory information continuously even during perturbations, rather than waiting to achieve steady target states for parameters such as force and velocity.

Blended rather than discrete movements minimize stress on the mechatronics of the robot and the object being handled. They can also take better advantage of the natural mechanics of interaction. For example, humans adjust grip and acceleration in parallel to prevent slip while minimizing grip force (Johansson and Flanagan, 2009). If a grasped object starts to slip as it is transported, possible corrective actions include adjustments to grip force (Veiga et al., 2018) as well as changes in translational acceleration and orientation with respect to gravity, all while considering the rate and direction of slip, the fragility of the object and the availability of intermediate support points near the current transport path. Those reactive strategies take years of practice to learn but fractions of a second to execute. They work because they blend smoothly—they fair—into the ongoing behavior rather than disrupting it with sudden state changes.

**FIGURE 5:**
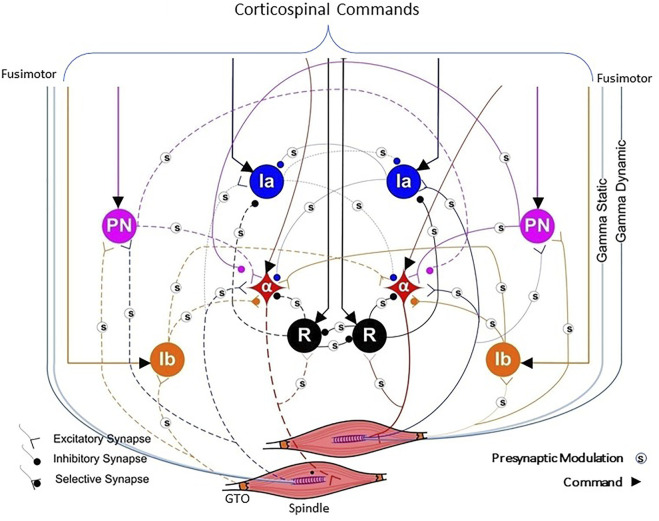
A highly simplified version of the typical spinal circuitry that mediates between commands from the cerebral cortex (bracketed lines indicating excitatory drive) and two muscles that could be used as either synergists or antagonists (e.g., wrist ulnar extensor and wrist radial extensor). Proprioceptive feedback arises from Golgi tendon organs (GTO) that sense force and muscle spindles whose sensitivity to length and velocity is independently modulated by the fusimotor gamma static and gamma dynamic neurons, respectively. The muscle fibers are controlled by alpha motoneurons (*α*) that provide inhibitory feedback *via* Renshaw cells (R). Other inhibitory interneurons are identified as Ia and Ib and excitatory propriospinal neurons (PN). An unknown number of the synapses are subject to presynaptic modulation (s) from other interneurons not explicitly depicted. Some of this is known to be driven by cutaneous receptors (Rudomin and Schmidt, 1999), suggesting a role in impedance control (Hogan, 1984) during dexterous manipulation. Adapted from (Raphael et al., 2010).

The components of the neuromusculoskeletal system that enable this fairing are remarkably unlike the corresponding components of current robots. They include the nonlinear mechanics of muscles and tendons (Tsianos and Loeb, 2017), the widespread distribution of cutaneous (Iggo, 1974) and proprioceptive sensors (Scott and Loeb, 1994; Mileusnic et al., 2006; Mileusnic and Loeb, 2009), and the convergence of multimodal feedback and command signals in the sophisticated interneuronal system of the spinal cord (Figure 5) (Pierrot-Deseilligny and Burke, 2005; Wolpaw, 2018). This has been modeled as a centrally programmable, multi-input-multi-output regulator (Loeb et al., 1990) that enables a wealth of interpolable programs that achieve graceful and energy efficient behaviors under normal and perturbed conditions (Raphael et al., 2010; Tsianos et al., 2011; Tsianos et al., 2014).

## 7 Bio-Inspired Development of Control Systems

The spinal cord (and presumably other subcortical integrative centers in midbrain and brainstem) provides a conceptual model for the robotic middleware that will be needed to mediate between discrete decision-making in the central controller (analogous to the mammalian cerebral motor cortex) and smooth execution by the robotic plant (see Figure 2). Unfortunately, the specific circuits of the biological spinal cord do not provide a working model for robotic middleware because the mechanical dynamics of robots tend to be unlike those of musculoskeletal systems. Achieving complementarity between a mechanical system and its controller is the well-known problem of system identification in engineering but a similar problem must already have been solved by biological systems. James Baldwin in 1896 pointed out that sudden changes in an organism’s environment and point mutations of its body form are the sine qua non of evolution but surviving them requires immediate behavioral adaptation by the organism. If the spinal circuitry were predetermined by the genetic transcriptome during cellular differentiation, a currently popular hypothesis (Osseward and Pfaff, 2019; Shin et al., 2020), then a similar problem would arise for the evolution of new species. The process of evolution itself thus favors organisms whose nervous systems are not hardwired and whose behavioral repertoires are learned (Baldwin, 1896; Baldwin, 1897; Partridge, 1982). When and how does all this adaptation and learning occur?

**FIGURE 6:**
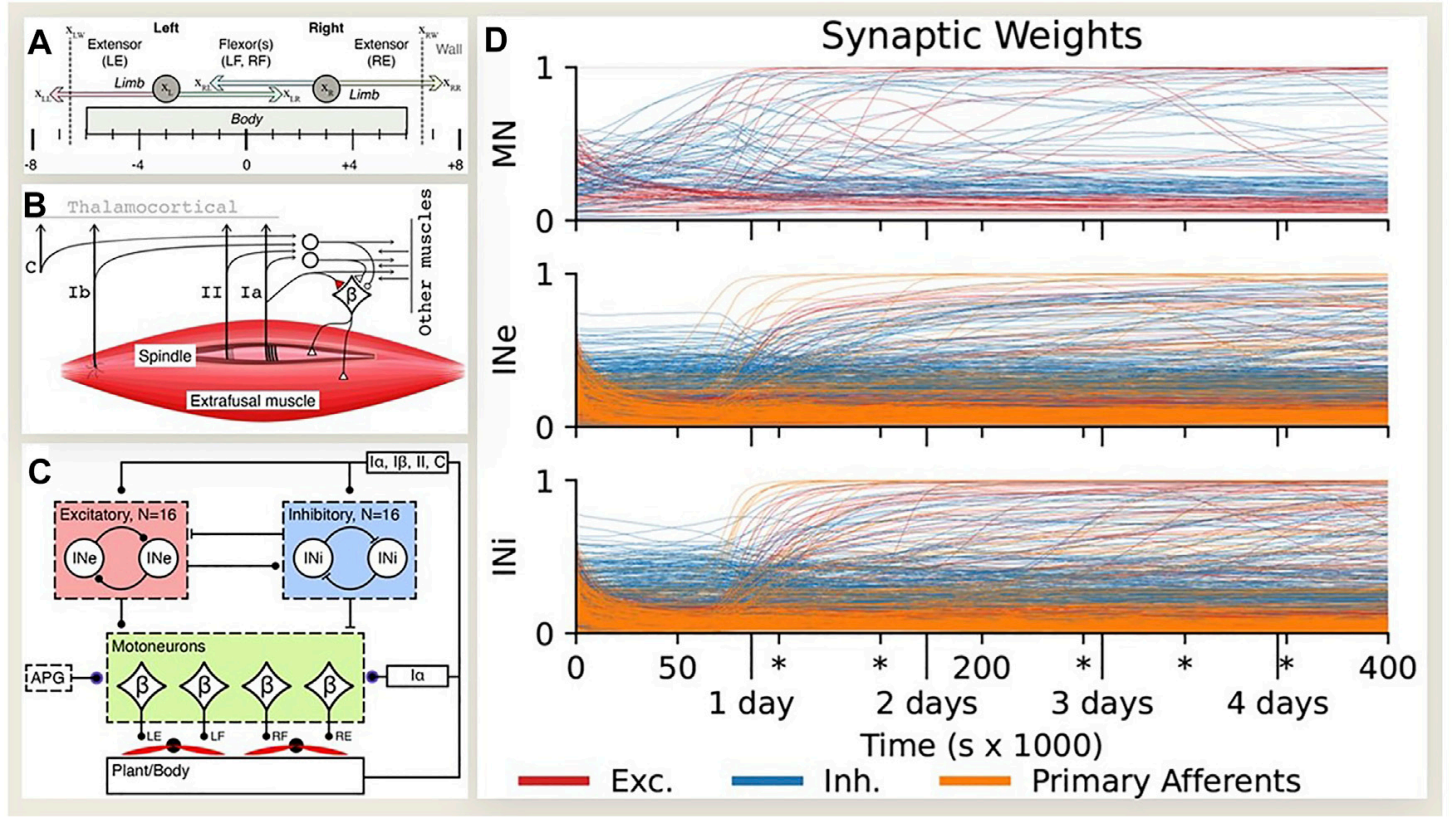
**(A)** Oropod model with two unidimensional limbs, each with two antagonist muscles. **(B)** Somatosensory receptors (C-cutaneous, Ib-Golgi tendon organ, II-spindle secondary, Ia-spindle primary) projecting onto beta motoneurons (βMNs) that have fusimotor collaterals. **(C)** Spinal neural network with 16 each excitatory (INe) and inhibitory (INi) interneurons receiving inputs with initially randomized gains from each other and all somatosensory afferents and projecting to all βMNs with randomized gains; direct inputs from Ia to βMNs; activity pattern generator (APG) of twitches in each muscle with randomized timing, amplitude and duration. **(D)** Development of input synaptic weights (color code at bottom) for each neuron type during simulated fetal development. After roughly a days’ worth of experience the initially random synaptic weights reorganize into mature and stable patterns, with only slow and sparse changes in the later stages. Redrawn from (Enander et al., 2022b), which provides detailed analysis of emergent patterns of connectivity that resemble those shown in Figure 5.

Experimental data (Petersson et al., 2003; Fagard et al., 2018) and modeling studies (Marques et al., 2013; Enander et al., 2022a; Enander et al., 2022b) suggest that details of the spinal connectivity may self-organize during spontaneous motor activity that occurs throughout fetal (Kiehn and Tresch, 2002) and perinatal development (Piek, 2006; Caligiore et al., 2008). If biological middleware self-organizes around the mechanics of the sensorimotor plant in which it finds itself, the same learning process might be applied to generate suitable middleware for arbitrary robotic plants (Blumberg et al., 2013). Enander et al. implemented simple Hebbian learning rules in initially randomized neural circuits connected to a model of a simplified musculoskeletal system (Figures 6A–C). Fetal-like random muscle twitches resulted in stable connectivity patterns (Figure 6D) that were similar to classical descriptions of proprioceptive spinal circuitry, including the homonymous monosynaptic stretch reflex, reciprocal length feedback, inhibitory force feedback and convergence of force and length feedback in excitatory premotor interneurons (Pierrot-Deseilligny and Burke, 2005; Alstermark et al., 2007). Similar motor babbling has been applied successfully to account for learned perception of body posture from physiological models of proprioceptors that project to the thalamocortical brain (Hagen et al., 2021). Cutaneous circuits are much less well characterized in animals but are known to be essential for dexterous manipulation, both to modulate grip forces and to trigger different phases of tasks (Johansson and Flanagan, 2009). Engineered multimodal tactile sensors are commercially available (Wettels et al., 2013) but their integration into control systems remains primitive and ad hoc. Self-organization might provide a basis for haptically enabled robots.

Biological development consists of many carefully orchestrated phases of growth and plasticity at various levels of the neuraxis (Hua and Smith, 2004); self-organizing robots will probably require something analogous (Weng et al., 2001; Der and Martius, 2017). Acquisition of a repertoire of motor actions proceeds in parallel with haptic characterization of self and world. It probably involves similarly incremental discovery through trial-and-error exploration (Loeb et al., 2011; Loeb, 2021) rather than the preprogrammed movements of most robots. The oft-invoked strategy of “bio-inspiration” for robotic design needs to be extended from the physical plant to every level of the control system. The design of those control systems should self-organize to reflect the dynamics of the plant rather than being preordained by arbitrary theories of control. Replicating these extended adaptive processes in robots further mandates the development of model-based training platforms.

## 8 Concluding Thoughts

Cognitive insights such as affordance appear to require at least some of the understanding of fundamental relationships that AI is still lacking. We have speculated, but not yet demonstrated, that adding a hierarchically abstractive architecture to the associational neural network for classifying objects according to causality (Figure 3) could enable the abstract and creative thought that has been most elusive for AI to date (Loeb and Fishel, 2014). Such hierarchies are in widespread use in perceptual neural networks (Hinton, 2012) and they have been extended to separately trainable modules for perception, policy and action (Hamalainen et al., 2019). Integrating them into physically embodied systems to control exploratory actions *via* middleware with sensory feedback will be challenging (Roy et al., 2021) but may be a necessary step toward truly intelligent machines.

Haptic robots provide an opportunity to update the original Turing test (Avraham et al., 2012) to reflect what has already been accomplished in AI and what remains to be done before robots can function alongside or in lieu of humans in the unstructured workplaces where humans thrive. Alan Turing aimed his AI aspirations toward the end of a century that was then at its midpoint. The next technological challenges outlined above are daunting but conceptually modest compared to those faced by electronic computing in 1950, still struggling with vacuum tubes and relays. Our technological armamentarium is now much richer and its rate of development much faster. Success in this endeavor promises intelligent robots that would address impending demographic crises of manpower in healthcare, manufacturing, agriculture and transportation. As they say in Silicon Valley, “Go big or go home.”

## References

[B1] Alstermark B. Isa T. Pettersson L.-G. Sasaki S. (2007). The C3?C4 Propriospinal System in the Cat and Monkey: a Spinal Pre-motoneuronal Centre for Voluntary Motor Control. Acta Physiol. 189 (2), 123–140. 10.1111/j.1748-1716.2006.01655.x 17250564

[B2] Avraham G. Nisky I. Fernandes H. L. Acuna D. E. Kording K. P. Loeb G. E. (2012). Toward Perceiving Robots as Humans: Three Handshake Models Face the Turing-like Handshake Test. IEEE Trans. Haptics 5 (3), 196–207. 10.1109/toh.2012.16 26964106

[B3] Bajcsy R. Aloimonos Y. Tsotsos J. K. (2018). Revisiting Active Perception. Auton. Robot. 42 (2), 177–196. 10.1007/s10514-017-9615-3 PMC6954017 31983809

[B4] Baldwin J. M. (1896). A New Factor in Evolution. Am. Nat. 30 (354), 441–451. 10.1086/276408

[B5] Baldwin J. M. (1897). Organic Selection. Science 5 (121), 634–636. 10.1126/science.5.121.634 17781159

[B6] Baum E. B. Moody J. Wilczek F. (1988). Internal Representations for Associative Memory. Biol. Cybern. 59 (4-5), 217–228. 10.1007/bf00332910

[B7] Bayes M. Price M. (1763). By the Late Rev. Mr. Bayes, F. R. S. Communicated by Mr. Price, in a Letter to John Canton, A. M. F. R. S. Philos. Trans. (1683-1775) 53, 370–418. (ArticleType: research-article/Full publication date: 1763/).

[B8] Bellman R. E. (1957). Dynamic Programming. Princeton University Press.

[B9] Blumberg M. S. Marques H. G. Iida F. (2013). Twitching in Sensorimotor Development from Sleeping Rats to Robots. Curr. Biol. 23 (12), R532–R537. 10.1016/j.cub.2013.04.075 23787051 PMC3709969

[B10] Bohg J. Hausman K. Sankaran B. Brock O. Kragic D. Schaal S. (2017). Interactive Perception: Leveraging Action in Perception and Perception in Action. IEEE Trans. Robot. 33 (6), 1273–1291. 10.1109/tro.2017.2721939

[B11] Bower G. H. (1970). Analysis of a Mnemonic Device: Modern Psychology Uncovers the Powerful Components of an Ancient System for Improving Memory. Am. Sci. 58 (5), 496–510.

[B12] Caligiore D. Ferrauto T. Parisi D. Accornero N. Capozza M. Baldassarre G. (2008). Using Motor Babbling and Hebb Rules for Modeling the Development of Reaching with Obstacles and Grasping. Int. Conf. Cognitive Syst., 22–23.

[B13] Coren S. Girgus J. (2020). Seeing Is Deceiving: The Psychology of Visual Illusions. England, UK: Routledge.

[B14] Der R. Martius G. (2017). Self-Organized Behavior Generation for Musculoskeletal Robots. Front. Neurorobot 11, 8. 10.3389/fnbot.2017.00008 28360852 PMC5352682

[B15] di Pellegrino G. Fadiga L. Fogassi L. Gallese V. Rizzolatti G. (1992). Understanding Motor Events: A Neurophysiological Study. Exp. Brain Res. 91, 176–180. 10.1007/bf00230027 1301372

[B16] Enander J. M. D. Jones A. M. Kirkland M. Hurless J. Jörntell H. Loeb G. E. (2022a). A Model for Self-Organization of Sensorimotor Function: The Spinal Monosynaptic Loop. J. Neurophysiology 127, 1460–1477. Epub ahead of print 2022 Mar 09. 10.1152/jn.00242.2021 35264006 PMC9208450

[B17] Enander J. M. D. Loeb G. E. Jörntell H. (2022b). A Model for Self-Organization of Sensorimotor Function: Spinal Interneuronal Integration. J. Neurophysiology 127, 1478–1495. Epub ahead of print 2022 Apr 27. 10.1152/jn.00054.2022 35475709 PMC9293245

[B18] Fagard J. Esseily R. Jacquey L. O'Regan K. Somogyi E. (2018). Fetal Origin of Sensorimotor Behavior. Front. Neurorobot 12, 23. 10.3389/fnbot.2018.00023 29875649 PMC5974044

[B19] Fishel J. A. Loeb G. E. (2012). Bayesian Exploration for Intelligent Identification of Textures. Front. Neurorobot 6 (4), 4. 10.3389/fnbot.2012.00004 22783186 PMC3389458

[B20] Fishel J. A. (2017). Personal Communication. Montrose, CA: SynTouch Inc.

[B21] French R. (1999). Catastrophic Forgetting in Connectionist Networks. Trends Cognitive Sci. 3 (4), 128–135. 10.1016/s1364-6613(99)01294-2 10322466

[B22] Gerstner W. (1990). Associative Memory in a Network Ofbiological'neurons. Adv. Neural Inf. Process. Syst. 3.

[B23] Gibson J. J. (1977). The Theory of Affordances. *Perceiving, Acting, and Knowing: Toward an Ecological Psychology* . Hillsdale, NJ: Lawrence Erlbaum Associates, 67–82.

[B24] Goodfellow I. J. Shlens J. Szegedy C. (2014). Explaining and Harnessing Adversarial Examples. Mach. Learn. 20, 1412. *arXiv preprint arXiv:1412.6572* . 10.48550/arXiv.1412.6572

[B25] Guo S. Shi Y. Yang Y. Xiao B. (2017). Energy Efficiency Maximization in Mobile Wireless Energy Harvesting Sensor Networks. IEEE Trans. Mob. Comput. 17 (7), 1524–1537. 10.1109/TMC.2017.2773067

[B26] Hagen D. A. Marjaninejad A. Loeb G. E. Valero-Cuevas F. J. (2021). insideOut: A Bio-Inspired Machine Learning Approach to Estimating Posture in Robots Driven by Compliant Tendons. Front. Neurorobot 15 (130), 679122. 10.3389/fnbot.2021.679122 34707488 PMC8542795

[B27] Halassa M. M. Sherman S. M. (2019). Thalamocortical Circuit Motifs: A General Framework. Neuron 103 (5), 762–770. 10.1016/j.neuron.2019.06.005 31487527 PMC6886702

[B28] Hamalainen A. Arndt K. Ghadirzadeh A. Kyrki V. (2019). “Affordance Learning for End-To-End Visuomotor Robot Control,” in IEEE/RSJ International Conference on Intelligent Robots and Systems (IROS). Affordance Learning for End-to-End Visuomotor Robot Control, 10 Mar 2019. 10.1109/iros40897.2019.8968596

[B29] Hazy T. E. Frank M. J. O'Reilly R. C. (2007). Towards an Executive without a Homunculus: Computational Models of the Prefrontal Cortex/basal Ganglia System. Phil. Trans. R. Soc. B 362 (1485), 1601–1613. 10.1098/rstb.2007.2055 17428778 PMC2440774

[B30] He H. Shang Y. Yang X. Di Y. Lin J. Zhu Y. (2019). Constructing an Associative Memory System Using Spiking Neural Network. Front. Neurosci. 13, 650. 10.3389/fnins.2019.00650 31333397 PMC6615473

[B31] Hebb D. O. (1949). The Organization of Behavior. New York: Wiley.

[B32] Held R. Hein A. (1963). Movement-produced Stimulation in the Development of Visually Guided Behavior. J. Comp. physiological Psychol. 56 (5), 872–876. 10.1037/h0040546 14050177

[B33] Hinton G. E. (2012). “A Practical Guide to Training Restricted Boltzmann Machines,” in Neural Networks: Tricks of the Trade. Editors Orr G. B. Müller Klaus-Robert (New York: Springer), 599–619. 10.1007/978-3-642-35289-8_32

[B80] Hogan N. (1984). An Organising Principle for a Class of Voluntary Movements. J. Neurosci. 4 (11), 2745–2754. 6502203 10.1523/JNEUROSCI.04-11-02745.1984 PMC6564718

[B34] Hua J. Y. Smith S. J. (2004). Neural Activity and the Dynamics of Central Nervous System Development. Nat. Neurosci. 7 (4), 327–332. 10.1038/nn1218 15048120

[B35] Hwangbo J. Lee J. Dosovitskiy A. Bellicoso D. Tsounis V. Koltun V. (2019). Learning Agile and Dynamic Motor Skills for Legged Robots. Sci. Robot. 4 (26), eaau5872. 10.1126/scirobotics.aau5872 33137755

[B36] Iggo A. (1974). “Cutaneous Receptors,” in The Peripheral Nervous System. Editor Hubbard J. I. (New York: Plenum Press), 347–404. 10.1007/978-1-4615-8699-9_14

[B37] Ivaldi S. Peters J. Padois V. Nori F. (2014). Tools for Simulating Humanoid Robot Dynamics: A Survey Based on User Feedback in IEEE-RAS International Conference on Humanoid Robots, Madrid, Spain, 18-20 November 2014 (IEEE), 842–849. 10.1109/HUMANOIDS.2014.7041462

[B38] Jamone L. Ugur E. Cangelosi A. Fadiga L. Bernardino A. Piater J. (2018). Affordances in Psychology, Neuroscience, and Robotics: A Survey. IEEE Trans. Cogn. Dev. Syst. 10 (1), 4–25. 10.1109/tcds.2016.2594134

[B39] Jie S. Moore J. L. Bobick A. Rehg J. M. (2010). Learning Visual Object Categories for Robot Affordance Prediction. Int. J. Robotics Res. 29 (2-3), 174–197. 10.1177/0278364909356602

[B40] Johansson R. S. Flanagan J. R. (2009). Coding and Use of Tactile Signals from the Fingertips in Object Manipulation Tasks. Nat. Rev. Neurosci. 10, 345–359. 10.1038/nrn2621 19352402

[B41] Katz D. (1925). Der aufbau der tastwelt. New York: Springer.

[B42] Kiehn O. Tresch M. C. (2002). Gap Junctions and Motor Behavior. Trends Neurosci. 25 (2), 108–115. 10.1016/s0166-2236(02)02038-6 11814564

[B43] Klatzky R. L. Lederman S. J. (2003). Touch Handbook of Psychology. Hoboken: John Wiley & Sons. 10.1002/0471264385.wei0406

[B44] Kudithipudi D. Aguilar-Simon M. Babb J. Bazhenov M. Blackiston D. Bongard J. (2022). Biological Underpinnings for Lifelong Learning Machines. Nat. Mach. Intell. 4 (3), 196–210. 10.1038/s42256-022-00452-0

[B45] Lansner A. (2009). Associative Memory Models: from the Cell-Assembly Theory to Biophysically Detailed Cortex Simulations. Trends Neurosci. 32 (3), 178–186. 10.1016/j.tins.2008.12.002 19187979

[B46] Läubli S. Castilho S. Neubig G. Sennrich R. Shen Q. Toral A. (2020). A Set of Recommendations for Assessing Human–Machine Parity in Language Translation. J. Artif. Intell. Res. 67, 653. 10.48550/arXiv.2004.01694

[B47] Loeb G. E. Fishel J. A. (2014). Bayesian Action&perception: Representing the World in the Brain. Front. Neurosci. 8, 341. 10.3389/fnins.2014.00341 25400542 PMC4214374

[B48] Loeb G. E. (2021). Learning to Use Muscles. J. Hum. Kinet. 76, 9–33. 10.2478/hukin-2020-0084 33603922 PMC7877274

[B49] Loeb G. E. Levine W. S. He J. (1990). Understanding Sensorimotor Feedback through Optimal Control. Cold Spring Harb. Symposia Quantitative Biol. 55, 791–803. 10.1101/sqb.1990.055.01.074 2132855

[B50] Loeb G. E. Tsianos G. A. Fishel J. A. Wettels N. Schaal S. (2011). Understanding Haptics by Evolving Mechatronic Systems. Prog. Brain Res. 192, 129–144. 10.1016/b978-0-444-53355-5.00009-9 21763523

[B51] Marques H. G. Imtiaz F. Iida F. Pfeifer R. (2013). Self-organization of Reflexive Behavior from Spontaneous Motor Activity. Biol. Cybern. 107 (1), 25–37. 10.1007/s00422-012-0521-7 23053431

[B52] Mcclelland J. L. (2013). Integrating Probabilistic Models of Perception and Interactive Neural Networks: a Historical and Tutorial Review. Front. Psychol. 4, 503. 10.3389/fpsyg.2013.00503 23970868 PMC3747375

[B53] Mileusnic M. P. Brown I. E. Lan N. Loeb G. E. (2006). Mathematical Models of Proprioceptors. I. Control and Transduction in the Muscle Spindle. J. Neurophysiology 96 (4), 1772–1788. 10.1152/jn.00868.2005 16672301

[B54] Mileusnic M. P. Loeb G. E. (2009). Force Estimation from Ensembles of Golgi Tendon Organs. J. Neural Eng. 6 (3), 036001. 10.1088/1741-2560/6/3/036001 19367000

[B55] Narang Y. Van Wyk K. Mousavian A. Fox D. (2020). Interpreting and Predicting Tactile Signals via a Physics-Based and Data-Driven Framework. Robotics: Science and Systems Foundation.

[B56] Newell D. Duffy M. (2019). Review of Power Conversion and Energy Management for Low-Power, Low-Voltage Energy Harvesting Powered Wireless Sensors. IEEE Trans. Power Electron. 34 (10), 9794–9805. 10.1109/tpel.2019.2894465

[B57] Osseward P. J. II Pfaff S. L. (2019). Cell Type and Circuit Modules in the Spinal Cord. Curr. Opin. Neurobiol. 56, 175–184. 10.1016/j.conb.2019.03.003 30954861 PMC8559966

[B58] Oztop E. Kawato M. Arbib M. A. (2013). Mirror Neurons: Functions, Mechanisms and Models. Neurosci. Lett. 540, 43–55. 10.1016/j.neulet.2012.10.005 23063951

[B59] Partridge L. D. (1982). The Good Enough Calculi of Evolving Control Systems: Evolution Is Not Engineering. Am. J. Physiology-Regulatory, Integr. Comp. Physiology 242, R173–R177. 10.1152/ajpregu.1982.242.3.r173 7065209

[B60] Petersson P. Waldenström A. Fåhraeus C. Schouenborg J. (2003). Spontaneous Muscle Twitches during Sleep Guide Spinal Self-Organization. Nature 424 (6944), 72–75. 10.1038/nature01719 12840761

[B61] Piek J. P. (2006). Human Motor Development. Champaign, IL: Human Kinetics 10.

[B62] Pierrot-Deseilligny E. Burke D. C. (2005). The Circuitry of the Human Spinal Cord: Its Role in Motor Control and Movement Disorders. Cambridge University Press.

[B63] Raphael G. Tsianos G. A. Loeb G. E. (2010). Spinal-Like Regulator Facilitates Control of a Two-Degree-Of-Freedom Wrist. J. Neurosci. 30 (28), 9431–9444. 10.1523/jneurosci.5537-09.2010 20631172 PMC6632449

[B64] Rosenblatt F. (1958). The Perceptron: A Probabilistic Model for Information Storage and Organization in the Brain. Psychol. Rev. 65 (6), 386–408. 10.1037/h0042519 13602029

[B65] Roy N. Posner I. Barfoot T. Beaudoin P. Bengio Y. Bohg J. (2021). From Machine Learning to Robotics: Challenges and Opportunities for Embodied Intelligence. Mach. Learn. *arXiv preprint arXiv:2110.15245* . 10.48550/arXiv.2110.15245

[B79] Rudomin P. Schmidt R. F. (1999). Presynaptic Inhibition in the Vertebrate Spinal Cord Revisited. Exp. Brain Res. 129, 1–37. 10550500 10.1007/s002210050933

[B66] Scott S. Loeb G. (1994). The Computation of Position Sense from Spindles in Mono- and Multiarticular Muscles. J. Neurosci. 14 (12), 7529–7540. 10.1523/jneurosci.14-12-07529.1994 7996193 PMC6576884

[B67] Shin M. M. Catela C. Dasen J. (2020). Intrinsic Control of Neuronal Diversity and Synaptic Specificity in a Proprioceptive Circuit. Elife 9. 10.7554/eLife.56374 PMC7467731 32808924

[B68] Tian H. Pouyanfar S. Chen J. Chen S. C. Iyengar S. S. (2018). “Automatic Convolutional Neural Network Selection for Image Classification Using Genetic Algorithms,” in IEEE international conference on information reuse and integration (IRI), Salt Lake City, 06-09 July 2018 (IEEE), 444–451. 10.1109/iri.2018.00071

[B69] Tsianos G. A. Goodner J. Loeb G. E. (2014). Useful Properties of Spinal Circuits for Learning and Performing Planar Reaches. J. Neural Eng. 11 (5), 056006. 10.1088/1741-2560/11/5/056006 25082652

[B70] Tsianos G. A. Loeb G. E. (2017). Muscle and Limb Mechanics. Compr. Physiol. 7 (2), 429–462. 10.1002/cphy.c160009 28333378

[B71] Tsianos G. A. Raphael G. Loeb G. E. (2011). Modeling the Potentiality of Spinal-like Circuitry for Stabilization of a Planar Arm System. Prog. Brain Res. 194, 203–213. 10.1016/b978-0-444-53815-4.00006-6 21867805

[B72] Turing A. M. (1950). I.-Computing Machinery and Intelligence. Mind LIX, 433–460. 10.1093/mind/lix.236.433

[B73] Ugur E. Nagai Y. Sahin E. Oztop E. (2015). Staged Development of Robot Skills: Behavior Formation, Affordance Learning and Imitation with Motionese. IEEE Trans. Auton. Ment. Dev. 7 (2), 119–139. 10.1109/tamd.2015.2426192

[B74] Valente A. Baraldo S. Carpanzano E. (2017). Smooth Trajectory Generation for Industrial Robots Performing High Precision Assembly Processes. CIRP Ann. 66 (1), 17–20. 10.1016/j.cirp.2017.04.105

[B75] Veiga F. Peters J. Hermans T. (2018). Grip Stabilization of Novel Objects Using Slip Prediction. IEEE Trans. Haptics 11 (4), 531–542. 10.1109/toh.2018.2837744 29994541

[B76] Weng J. McClelland J. Pentland A. Sporns O. Stockman I. Sur M. (2001). Autonomous Mental Development by Robots and Animals. Science 291 (5504), 599–600. 10.1126/science.291.5504.599 11229402

[B77] Wettels N. Fishel J. Loeb G. (2013). “Multimodal Tactile Sensor,” in The Human Hand as an Inspiration for Robot Hand Development. Editors Balasubramanian R. Santos V. (New york: Springer).

[B78] Wolpaw J. R. (2018). The Negotiated Equilibrium Model of Spinal Cord Function. J. Physiol. 596 (16), 3469–3491. 10.1113/jp275532 29663410 PMC6092289

